# Bridging the Gap Between Language and Literacy: Evidence from Interventions in Young Greek-Speaking Children with Developmental Language Disorder

**DOI:** 10.3390/bs16050809

**Published:** 2026-05-18

**Authors:** Angeliki Mouzaki, Vasiliki Desylla, Asimina M. Ralli, Maria Vlassopoulos

**Affiliations:** 1 Primary Education Department, School of Education, University of Crete, Gallos Campus, 741 00 Rethymno, Crete, Greece; vasiliki.desylla@yahoo.com; 2 Department of Psychology, School of Philosophy, National and Kapodistrian University of Athens University Campus, 157 84 Ilisia, Greece; asralli@psych.uoa.gr; 3 Department of Medicine, School of Health Sciences, National and Kapodistrian University of Athens, Goudi, 115 27 Athens, Greece; vlassopoulosm@gmail.com

**Keywords:** developmental language disorder, language intervention, phonological awareness, vocabulary development

## Abstract

Developmental language disorder (DLD) is a neurodevelopmental condition characterized by persistent difficulties in language acquisition, affecting both comprehension and production, and typically emerging in early childhood through deficits in morphosyntax, vocabulary and phonology. Although distinct from Specific Learning Disabilities, particularly in reading, the two conditions often co-occur, sharing underlying mechanisms and leading to overlapping challenges such as impaired phonological processing, limited vocabulary, weak narrative skills, and reading comprehension difficulties. This study examined the effects of two intervention programs—semantic versus phonological—on oral language skills in 107 Greek children with DLD aged 4;1–5;10. Participants were randomly assigned to a phonological (*n* = 35), a semantic (*n* = 35), or a control group (*n* = 37). Interventions were delivered individually twice weekly over 16 weeks (32 sessions). Language performance was assessed at baseline, immediately post intervention, and at a four-month follow-up using standardized measures. Repeated-measures ANOVAs evaluated within- and between-group differences. Results indicated differential but complementary effects of the two interventions: phonological training enhanced greatly phonological awareness (d = 0.80) and was associated with short-term gains in vocabulary, whereas the semantic intervention produced sustained improvements in vocabulary (d = 0.45). While the semantic group performed slightly better than the control group, no statistically significant difference was found between the two intervention groups, suggesting broadly comparable but domain-specific benefits. These findings highlight the value of systematic interventions and indicate that combining semantic and phonological approaches may optimize language and literacy development, providing evidence-based guidance for early intervention in preschool children with DLD.

## 1. Introduction

The relation between oral language and literacy development has been extensively examined regarding its nature and strength, producing different interpretations of the evident connection between the two. A substantial body of evidence considers oral language as the foundation of literacy acquisition, while the interaction of language effects and familial and environmental factors sheds new light on the interpretation of literacy outcomes (Dandache et al., 2014; Hulme et al., 2015; Lervåg et al., 2018; Storch & Whitehurst, 2002; van Viersen et al., 2018).

Oral language, particularly phonological processing—the ability to identify, retain, and manipulate the sound units of spoken language such as syllables and phonemes—and vocabulary, plays a critical role in reading development, influencing decoding, word recognition, and reading comprehension in ways that shift across developmental stages. Research evidence consistently underscores this role, showing, from the earlier longitudinal studies, that early language skills predict later phonological awareness and letter knowledge, which, in turn, predict reading (NICHD Early Child Care Research Network, 2005; Storch & Whitehurst, 2002). Subsequent studies extended these findings by examining both the direct and indirect effects of early language on literacy outcomes, including the role of mediating preliteracy skills (i.e., rapid automatized naming) and also considering children with language vulnerabilities or familial risk (Duff et al., 2015; Hjetland et al., 2019; Hulme et al., 2015; Torppa et al., 2010).

Specifically, a longitudinal investigation with Finnish children provided a detailed account of how early receptive and expressive language at 2.5 years indirectly predicts word-reading ability at age 9, primarily through its impact on preliteracy skills measured at 3.5 and 5.5 years (Torppa et al., 2010). Similarly, Duff et al. (2015), in a two-wave study of British children, found that vocabulary knowledge between 16 and 24 months predicted later vocabulary, phonological awareness, reading accuracy, and reading comprehension at ages 5 to 9. Although family risk status was associated with reading accuracy and comprehension, it did not predict language outcomes.

In another longitudinal study following a cohort of British children from 3.5 to 8 years of age, Hulme et al. (2015) examined the developmental pathways from oral language and word reading to reading comprehension. Their findings indicated that early language skills at 3.5 years and word-level literacy at 5.5 years each contributed uniquely to reading comprehension. Early language appeared to exert both an indirect influence, mediated through preliteracy skills and word decoding, and a direct effect on reading comprehension, suggesting an independent language-based pathway to reading comprehension. Notably, these developmental patterns were consistent across children with and without a family risk of dyslexia. Language, as a unitary factor, was also found to influence reading comprehension in a stable manner during a 6-year longitudinal study of Norwegian children from age 4 years (Hjetland et al., 2019).

Such findings stress the critical role of early language development in laying the foundation for literacy acquisition and the need to address children’s language difficulties prior to the beginning of formal schooling. Children with language problems are widely considered as being at high risk for later learning disabilities, particularly in reading, and lower academic achievement (e.g., Bishop et al., 2017; Catts et al., 2008). Given this well-established link between early language skills and later literacy outcomes, increasing research attention has focused on children who experience language problems from early on, raising questions about the persistence of these challenges and their impact on learning. Within this context, the development and empirical evaluation of language interventions—particularly for children with developmental language disorder (DLD) is of particular importance, as early intervention may help mitigate language difficulties and potentially reduce the risk of later reading disorders.

### 1.1. Developmental Language Disorder (DLD)

Developmental language disorder (DLD) is a common neurodevelopmental condition that persists across the lifespan, affecting academic achievement, social functioning, and emotional development (Clegg et al., 2021). It is characterized by persistent difficulties in language processing that affect comprehension and/or production, without being attributable to sensory, cognitive, or neurological conditions (Bishop et al., 2017). Symptoms can involve phonology, grammar, vocabulary, and pragmatic language use, often varying in severity and presentation. Children with comprehension deficits generally show poorer prognosis and greater resistance to intervention (Law et al., 2003). The heterogeneity of the disorder is further shaped by comorbidities (speech sound disorder, dyslexia, ADHD, etc.) and overlapping linguistic processes (Bishop et al., 2017).

According to the most used diagnostic frameworks (DSM-5, ICD-11), and the CATALISE consortium (Bishop et al., 2017), DLD significantly limits communication, academic performance, and participation in daily life, with symptoms evident from early childhood and persisting into adulthood (American Psychiatric Association, 2022; Conti-Ramsden, 2008; World Health Organization, 2007). The CATALISE project further emphasized clinical risk markers and patterns of comorbidity over exclusionary criteria (Bishop et al., 2017). Epidemiological studies consistently report prevalence rates around 7% in early childhood (Tomblin et al., 1996; Norbury et al., 2016), underscoring the likelihood that in an average classroom two children may present with DLD.

### 1.2. DLD and Reading Disorder (Dyslexia)

A strong body of research has examined the association between DLD and reading disorder (dyslexia) given its pervasive impact on learning. Spoken language difficulties hinder the acquisition of literacy skills, with adverse consequences for school achievement (Botting, 2020; Snowling & Hulme, 2021; Ziegenfusz et al., 2022).

The relationship between DLD and dyslexia has been conceptualized in three main perspectives: (a) the severity model, (b) the additional deficit model, and (c) multifactorial or component models. The severity model proposes that both disorders stem from a common phonological deficit that affects early oral language and later reading development. Even though this perspective considers one disorder, it acknowledges that phonological deficits are more severe in DLD (Kamhi & Catts, 1986).

The additional deficit model suggests that, although both disorders share phonological processing impairments, their phenotypes differ: DLD is associated with broader deficits in semantics (knowledge of word meanings and conceptual relationships) and morphosyntax (the rules that govern word inflections and sentence structure) which can further affect reading comprehension and written language (Bishop & Snowling, 2004; A. M. Ralli & Palikara, 2013). In contrast, dyslexia is typically restricted to phonological deficits primarily affecting decoding and spelling (Bishop et al., 2017).

More recently, multifactorial or component models have also been proposed to capture these overlapping yet distinct profiles, adopting a dimensional rather than a categorical approach. These models suggest that the manifestation of DLD and dyslexia reflects different combinations of linguistic, cognitive, genetic and environmental risk factors. Within this perspective, phonological deficit may arise from partially dissociable profiles of phonological impairment among DLD and children with dyslexia. The weaknesses are described across two dimensions—phonological processing skills and phonological representations (i.e., the precision and stability of mental representations of speech sounds in long-term memory) (Ramus et al., 2013).

Research evidence with Greek-speaking children confirms this complexity. Talli et al. (2016) compared children with DLD and dyslexia to peers matched for age and reading level, finding both shared and distinct patterns across phonological awareness, decoding, and comprehension. In a more recent study, with children at the early stages of literacy acquisition the most evident deficits for children with DLD were oral language (phonological measures, listening comprehension, vocabulary and morphological awareness) and lower performance on verbal short-term memory while children at risk for dyslexia had more pronounced deficits in word decoding (Chalikia et al., 2025).

These findings stress the need for comprehensive assessment to delineate phenotypes. Current diagnostic frameworks (DSM-5) recognize DLD and dyslexia as distinct yet frequently co-occurring disorders, with dual diagnosis warranted when both significantly affect learning outcomes.

### 1.3. Language Interventions

As mentioned earlier, it is well documented that phonological deficits are strongly linked to poor decoding, whereas weaknesses in vocabulary, grammar, and receptive language more strongly affect reading comprehension (Snowling & Hulme, 2012). Moreover, children with persistent speech and language impairments at the school-entry time are at substantially increased risk for subsequent literacy difficulties, emphasizing the need for structured and systematic intervention (Rose, 2006). Even though many children with language difficulties eventually succeed in resolving their difficulties by the time they go to school and do not face problems in learning to read, they continue to present risk for subsequent problems with reading comprehension (Snowling et al., 2016, 2020; Thompson et al., 2015).

The need for early language interventions to prevent subsequent problems in reading and understanding written language has been consistently emphasized (Fricke et al., 2013; Hagen et al., 2017; Rogde et al., 2016). However, the heterogeneity of the clinical profile of children with DLD is also shaped by the influence of individual, familial, and comorbid factors. Individual cognitive characteristics, such as phonological processing abilities, working memory capacity, and processing speed, may affect how efficiently children process linguistic information. Familial influences, including genetic liability and the quality of the home language environment, may further shape early language development and literacy exposure. In addition, comorbid developmental conditions—such as attention difficulties or speech sound disorders—can interact with underlying language vulnerabilities, contributing to variability in both the severity of language difficulties and responsiveness to intervention. Together, these interacting influences help explain the wide variability observed in language and literacy outcomes and the corresponding diversity of intervention approaches, while also emphasizing the need for interventions grounded in evidence-based practice.

Research examining language interventions for vocabulary difficulties in children with developmental language disorder (DLD) has increased in recent years, yet there remains no clear consensus regarding the relative effectiveness of phonological and semantic approaches (Munro et al., 2008). Most intervention studies combine phonological and semantic components (e.g., Best et al., 2015, 2021; Bragard et al., 2012; Easton et al., 1997; German, 2002; Gray, 2005; Munro et al., 2008; A. M. Ralli & Dockrell, 2010), and relatively few have examined phonological interventions in isolation (Best, 2005; German, 2002). An interesting theoretical hypothesis that has not yet been tested with children with DLD is whether strengthening semantic knowledge could influence the phonemic representations of spoken words, as proposed by the Lexical Restructuring Hypothesis (LRH). Specifically, according to the LRH, word learning during development leads to increasingly detailed phonemic representations, which help children differentiate among the growing number of phonologically similar items in their spoken vocabularies (Fowler, 1991; J. M. Walley, 1998; A. C. Walley et al., 2003). Within this framework, interventions that enrich lexical–semantic knowledge may indirectly facilitate the refinement of phonological representations by promoting restructuring within the mental lexicon, a possibility that may be particularly relevant for children with DLD. Most recent semantic interventions aim to strengthen semantic networks through activities such as thematic and taxonomic categorization, semantic cueing, and explicit discussion of word meanings, synonyms, antonyms, and conceptual relations. These interventions have been implemented in a variety of contexts (e.g., school-based pull-out sessions, small groups, individual therapy, etc.) and with varying intensity, typically ranging from 16 to 25 sessions, although both shorter and longer programs have also been reported.

Evidence from English-speaking populations suggests that language interventions targeting vocabulary difficulties are generally beneficial for children’s language development, regardless of whether they adopt semantic or phonological approaches. Research comparing both approaches suggests that children respond differently depending on their linguistic profiles: children who make semantic errors tend to benefit more from semantic interventions, whereas those with phonological errors show greater gains from phonological training (Best et al., 2015, 2021). Semantic interventions appear to support word comprehension and conceptual connections, while phonological approaches may facilitate word production and lexical access strategies (Gray, 2005; German, 2002). Additional findings indicate that factors such as word characteristics, prior vocabulary knowledge, and age can influence outcomes, and that more holistic interventions incorporating multiple linguistic components may yield stronger effects (Porta & Ramirez, 2020).

Notably, there is currently no empirical evidence on the effectiveness of semantic or phonological interventions for Greek-speaking children. The existing literature lacks systematic investigations of intervention approaches, despite the considerable theoretical interest in examining intervention effects in languages such as Greek, which are characterized by rich inflectional and derivational morphology and relatively transparent phonology (A. Ralli, 2021).

The present study aims to address this gap by examining two early language intervention programs for 4–6-year-old children with DLD, one focusing on the development of phonological skills and the second focusing on the development of semantic knowledge and processing ability. The relative effectiveness of each program was assessed at two time points (immediately after completion and at a four-month follow-up) on two primary outcomes: phonological and semantic test scores.

Specifically, the study addressed the following research questions:

(1) Does each intervention program improve phonological and semantic skills in children with DLD?
(2) Do the two intervention programs differ in their effects on phonological and semantic outcomes?
(3) Are the effects of each intervention maintained four months after the completion of the program?

## 2. Methods

Participating children were randomly assigned to one of two individually delivered interventions within their schools by specially trained research assistants. Both interventions included 32 sessions that were scheduled biweekly in two phases lasting 8 weeks each with a 4 week break in between. All children were assessed three times during a 10-month period: prior to the intervention (T1), following completion of the intervention or at five months post T1 for the control group (T2), and 4 months after the end of the intervention (T3; intervention groups only). The study timeline is described in Table 1.

### 2.1. Participants

Participating children (*n* = 107) were selected among students attending Pre-K and Kindergarten in the town of Rethymno, Crete, between 2018 and 2022. In Greece, preschool education is mandatory, lasts two years, and serves children aged 4–6 years. The national preschool curriculum provides the regulatory framework for early childhood education and aims to promote children’s holistic development with emphasis on competencies such as communication, collaboration, creativity, and critical thinking through integrated thematic domains. One of these domains, “Child and Communication,” focuses on the development of oral language and children’s initial encounters with reading and writing. Within this framework, children are exposed to rich language experiences through shared book reading, narrative activities, and multimodal text exploration, while also developing early literacy-related skills such as vocabulary, phonological awareness, emerging knowledge of letter–sound correspondences, and early writing attempts.

Inclusion criteria for the study participants were the following: (i) age 4–6 years, (ii) the child’s first language was Greek, (iii) both parents had completed at least secondary education, (iv) presence of language delay as indicated by score < 50o percentile on the index of general language proficiency derived from a standardized language test, Logometro (Mouzaki et al., 2017), (v) score < −1.25 SDs below average on any two language domains (i.e., receptive/expressive vocabulary, phonological, morphological, narrative, etc.), and (vi) typical non-verbal intelligence as indicated by a standard score >80 on Raven’s Colored Progressive Matrices (Raven, n.d.; Sideridis et al., 2015). Exclusion criteria were diagnosis of any other developmental disorder and/or sensory deficit as well as participation in speech therapy services, currently or in the past.

All students between 4 and 6 years of age with positive parental consent participated in the screening procedure for language skills and non-verbal intelligence. Students who met all criteria for study participation were randomly allocated to three groups without regard to initial pre-test score status to minimize allocation bias, while maintaining approximate gender balance across groups (details are summarized in Table 2). Baseline equivalence between groups was subsequently examined using the pre-test measures. As indicated in Table 2, all groups were closely comparable on non-verbal IQ, phonological and semantic skills. After completing the second assessment, children in the control group were offered one of the two intervention programs for ethical reasons, to ensure that all participants had access to potentially beneficial instruction. Importantly, this delayed intervention was implemented only after the second assessment had been completed and did not influence the comparisons between groups at post-test or the follow-up analyses reported in the present study.

### 2.2. Measures

#### 2.2.1. Non-Verbal Intelligence

The Greek standardization of the Raven’s Colored Progressive Matrices (Raven, n.d.; Sideridis et al., 2015) was used to assess non-verbal intelligence. Cronbach’s alpha reliability coefficient has been reported to be 0.90 (Sideridis et al., 2015).

#### 2.2.2. Language Assessments

Logometro (Mouzaki et al., 2017) was used to assess language skills. It is a standardized Greek-language assessment battery designed to assess a broad range of language skills in children aged 4–7 years that is administered through a tablet-based Android application for mobile devices. The digital administration enables proper voicing of directions, easy capturing of children’s responses via touch screens and direct recordings (for oral replies). Logometro’s standardization sample consisted of 800 children (384 boys, 416 girls) between 4 and 7 years of age drawn from a total pool of 926 participants in the larger validation study. Age was stratified into six groups using 6-month intervals ranging from 4;0 to 6;11 years, with approximately 100–104 participants per group and balanced gender representation. The normative sample included only typically developing children and excluded those with diagnoses of developmental language disorder (DLD), sensory impairments, or other developmental delays. The psychometric properties of the assessment battery have been documented in prior research (Antoniou et al., 2022; Mouzaki et al., 2020). Logometro assesses the following domains: phonological awareness, oral language comprehension, receptive and expressive vocabulary, narrative skills, morphological awareness, pragmatic competence, and emergent literacy skills. Each administered task consists of two practice items that include feedback and could be repeated if necessary, and items ordered by increasing difficulty, with floor and ceiling rules. Responses are scored automatically as the number of correct answers.

#### 2.2.3. Phonological Skills

For assessing phonological awareness skills Logometro includes eight developmentally appropriate tasks for preschool-aged children: initial syllable identification (Cronbach’s α = 0.84), initial phoneme identification (α = 0.84), syllabic synthesis (α = 0.89), phonemic synthesis (α = 0.93), syllabic segmentation (α = 0.95), phonemic segmentation (α = 0.95), syllabic deletion (α = 0.94), and phoneme deletion (α = 0.92). All tasks contained seven items, scored with 1 or 0, and administration was discontinued following four consecutive errors. Logometro calculates a single index by averaging performance on the eight tasks converted to age-referenced scores based on the normative data.

#### 2.2.4. Semantic Skills

Semantic skills were assessed with three different Logometro tasks, Receptive vocabulary (α = 0.88), Expressing vocabulary (α = 0.93) and Naming (α = 0.72), which provided a single score for semantic knowledge (Mouzaki et al., 2017). Test items/words in all three tasks were in ascending order of difficulty, were scored with 1 or 0, and the administration of each task was stopped when children made a number of consecutive errors according to each task’s ceiling criterion. Language tests were administered in a fixed order, individually to each child in one or two 40 min sessions in a quiet room at their schools by the second author.

### 2.3. Intervention Procedures

The two intervention programs were developed especially for the study purposes by the second author and consisted of 32 30 min sessions. Both programs were based upon the same word materials that were selected after detailed examination of similar programs, the Greek preschool curriculum (Penteri et al., 2021), and other relevant resources. The word categories addressed included body parts, clothing, food items, toys, household objects, school-related objects, farm animals, and wild animals. The first phase of the semantic program focused on acquisition of new vocabulary words, word categorizations based on thematic and taxonomic relations, and finally, on establishing semantic associations and metacognitive awareness. The second phase of the semantic program included activities involving semantic analogies, sentence completion, and descriptions/definitions, as well as narrative production.

During the first phase of the phonological program children were encouraged to practice syllable awareness, to identify rhymes in words, to isolate and compare syllables in words, to segment words into syllables and to combine them to form words. During the second phase of the program children were provided with ample opportunities to practice with phonemes in the words. The intervention programs were implemented as structured, play-based instructional sessions delivered individually. Each session included a sequence of brief, highly interactive activities targeting specific skills using picture cards, worksheets and little objects. Tasks were presented within game-like scenarios to sustain motivation. Activities followed a consistent instructional structure including explicit task instructions, guided practice, and immediate corrective and supportive feedback, with additional scaffolding provided when children experienced difficulty with the task. A more detailed content description of both programs and task examples are provided in Table S1 in the Supplementary Materials.

To minimize the potential impact of distractions associated with the public-school setting, all intervention activities were implemented in the same type of setting across groups (quiet room within school). Occasional interruptions (e.g., recess, school celebrations, or classroom activities) applied equally to both intervention groups and were therefore unlikely to differentially influence outcomes.

Children in the control group continued to participate in their regular classroom instruction provided by their schools (business as usual). For ethical reasons, children in the control group were offered one of the two intervention programs after they completed the second assessment.

To ensure reliable delivery of both programs, a manual was developed for each intervention and each research assistant underwent thorough training and evaluation for its use prior to program delivery. Also, to ensure fidelity, all assistants completed a special form in each session with the child’s responses and other information, and in addition they were observed every 10 sessions.

### 2.4. Statistical Analysis

Mixed-model ANOVAs were used to address the first research question with time (T0, T1) as a repeated-measures variable and group (semantic intervention, phonological intervention, control) as a between-subjects variable. A separate mixed ANOVA was conducted to examine whether the long-term effects of each intervention (i.e., at the 4-month follow-up) varied between the two intervention groups. Main effects and interactions were assessed at Bonferroni-adjusted *p* = 0.05/2 = 0.025. Significant interactions were followed up by tests of simple main effects of group at each time point and of time within each group (evaluated at *p* = 0.05/3 = 0.017 to control for inflated family-wise Type I error). Effect sizes were expressed as Cohen’s d. Within-group (repeated-measures) effects were estimated using the dav metric (Lakens, 2013), which standardizes the mean change using the average standard deviation of the two time points and incorporates the correlation between repeated measurements for confidence interval estimation. Between-group effects were calculated using Cohen’s d based on the pooled standard deviation of the two groups, with 95% confidence intervals derived from the standard error of the standardized mean difference. Analyses were performed using SPSS version 20.0 (IBM Corp., Armonk, NY, USA).

## 3. Results

### 3.1. Group Effects at T0 and T1

*Phonological Awareness.* The three groups performed comparably at baseline on the Phonological Index (simple main effect of group: *p* = 0.9) (see Table 3). 

**Figure 1:**
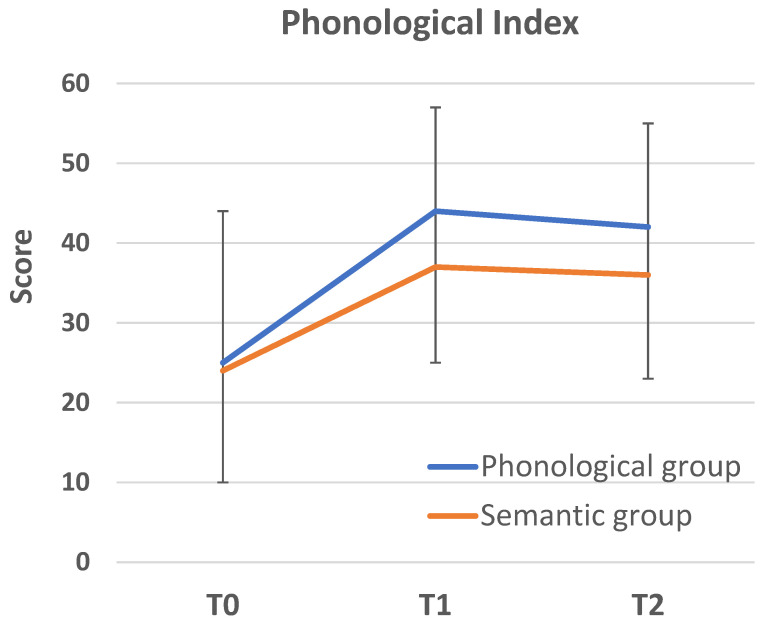
Average score on the Phonological Index measured at three time points (intervention groups) and at two time points in the control group. Vertical bars represent SD values.

As indicated by a significant time by group interaction on the Phonological Index, F(2,104) = 6.43, *p* = 0.002, η^2^ = 0.11, the degree of improvement in phonological awareness varied across groups (see Figure 1 and Table 4), in the absence of a group main effect (*p* = 0.16), although the main effect of time was also significant, F(1,104) = 30.90, *p* < 0.001, η^2^ = 0.23. Following the significant interaction, simple main effects were examined. At T1 the simple main effect of group was significant, F(1,104) =5.45, *p* = 0.006, η^2^ = 0.10, and pairwise comparisons revealed that the phonological group outperformed the control group (*p* = 0.001) with a large effect size (Cohen’s d = 0.80, 95% confidence interval [CI] = 0.24–1.28) (all post hoc comparisons were evaluated against the Bonferroni-adjusted threshold of *p* < 0.017). No statistically significant differences were observed between the semantic group and either the control group (*p* = 0.068) or the phonological group (*p* = 0.15). Although these differences were not statistically significant and should be interpreted with caution, the associated effect sizes were in the medium range (semantic vs. control: d = 0.45, 95% CI = 0.11–0.89; semantic vs. phonological: d = 0.40, 95% CI = 0.08–0.84).

As revealed by repeated-measures simple main effects tests, both the phonological, F(1,34) = 25.55, *p* < 0.001, η^2^ = 0.44, and semantic groups, F(1,34) = 11.58, *p* = 0.002, η^2^ = 0.25, showed significant improvement on the Phonological Index, although performance change did not approach significance in the control group (*p* = 0.5, d = 0.08, 95% CI = −0.22–0.38). However, the degree of improvement represented a large effect size in the phonological group (d = 0.87, 95% CI = 0.60–1.34) and only a medium effect size in the semantic group (d = 0.57, 95% CI = 0.25–0.96).

**Figure 2:**
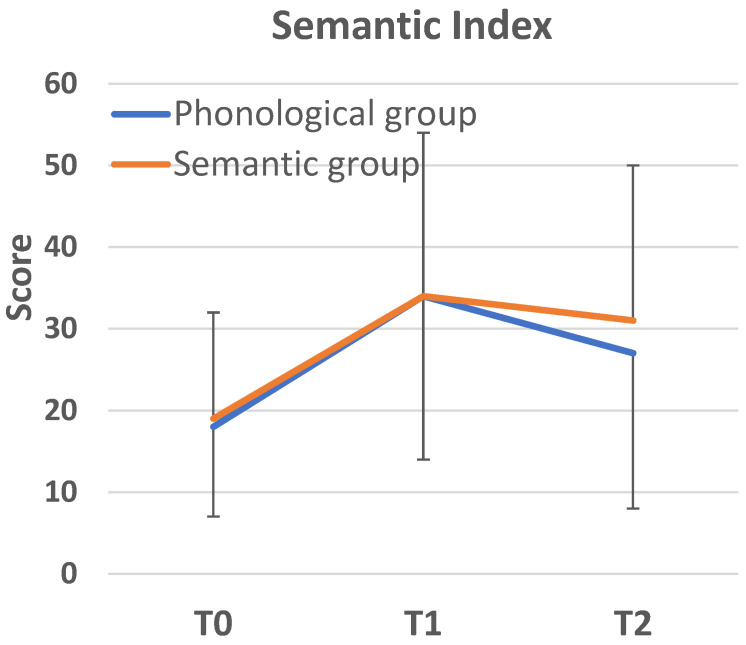
Average score on the Semantic Index measured at three time points (intervention groups) and at two time points in the control group. Vertical bars represent SD values.

*Semantic Skills.* As indicated by a significant time by group interaction on the Semantic Index, F(2,104) = 5.55, *p* = 0.005, η^2^ = 0.097, the degree of improvement in semantic knowledge varied across groups (see Figure 2 and Table 5). It should be noted that the group main effect was not significant (*p* = 0.08), although the main effect of time was, F(1,104) = 51.66, *p* < 0.001, η^2^ = 0.32. Following the significant interaction, simple main effects were examined. These tests revealed that the three groups performed comparably at baseline (main effect of group: *p* = 0.9). At T1 the simple main effect of group was significant, F(1,104) = 4.76, *p* = 0.011, η^2^ = 0.08, and pairwise comparisons revealed that the semantic group outperformed the control group (*p* = 0.007, with a moderately large effect size: d = 0.66, 95% CI = 0.14–1.17). Performance of the phonological group was also significantly higher than performance of the control group (*p* = 0.011, with a moderately large effect size: d = 0.62, 95% CI = 0.11–1.13), yet it did not differ from the average score of the semantic group (*p* = 0.9, d = 0.05, 95% CI = −0.43–0.50 [very small]). All post hoc comparisons were evaluated against the Bonferroni-adjusted threshold of *p* < 0.017).

As revealed by repeated-measures simple main effects tests, both the phonological, F(1,34) = 20.55, *p* < 0.001, η^2^ = 0.37, and semantic groups, F(1,34) = 38.02, *p* < 0.001, η^2^ = 0.53, showed significant improvement on the Semantic Index, although performance change did not reach significance in the control group (*p* = 0.15). The degree of improvement represented a large effect size for both the phonological group (d = 0.76, 95% CI = 0.45–1.19) and the semantic group (d = 0.88, 95% CI = 0.56–1.21).

### 3.2. Retention of Intervention Effects

**Figure 3:**
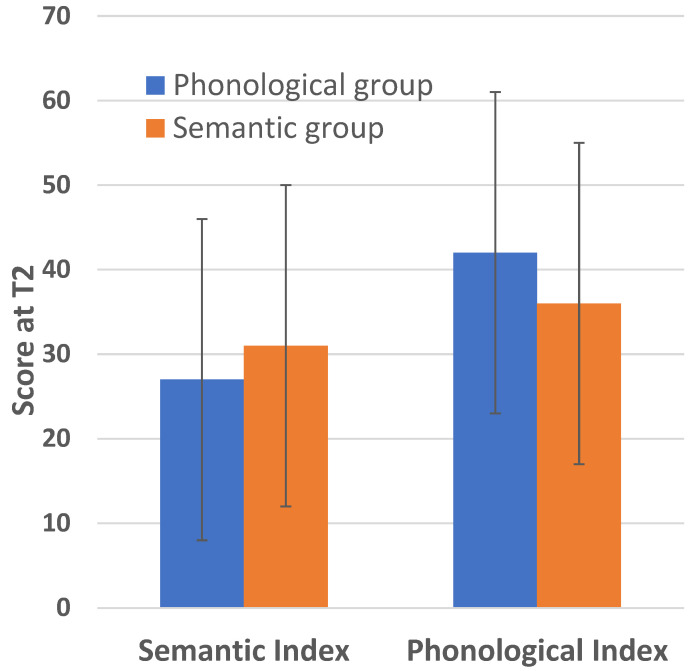
Average score on the Phonological and Semantic Indices measured at the 4-month follow-up for the groups that received the phonological and semantic intervention. Vertical bars represent SD values.

The mixed-model ANOVA with group (phonological, semantic) as the between-subjects variable and type of skill measured at the 4-month follow-up as the within-subjects variable revealed a significant interaction, F(1,68) = 5.587, *p* = 0.018, η^2^ = 0.079 (see Figure 3), in the absence of a group main effect (*p* = 0.8), supporting the notion that the differential effects of the intervention on each target skill persisted for some time after the completion of the intervention.

## 4. Discussion

This study aimed to compare two language intervention programs for children with DLD, in order to examine their effectiveness in supporting two skills that are critical for literacy development, namely phonemic awareness and vocabulary. Given the well-established link between oral language abilities and subsequent reading development, a focus on effective language interventions may be relevant not only for language development but also for later literacy outcomes.

The first two research questions assessed and contrasted the effects of these programs on enhancing the corresponding skills in children with DLD aged 4 to 6 years. The third research question examined whether any gains were maintained four months after the completion of the intervention. By evaluating and comparing their impact on phonological and semantic skills of children aged 4 to 6 years, the study adds to the emerging evidence base on language interventions for young children with DLD and provides insights into how different linguistic approaches may support language development in this population.

Both interventions were implemented with experimental groups and compared to a control group. Post-tests and delayed re-testing confirmed domain-specific gains: phonological training increased phonological awareness and yielded short-term vocabulary benefits, while semantic-focused intervention supported durable vocabulary growth.

### 4.1. Phonological Intervention

Participants in the phonological intervention group practiced auditory identification and manipulation of syllables and phonemes, supported by picture-based materials. Consistent with what was anticipated, the results revealed statistically significant improvements in phonological awareness tasks at both the second and third assessments. These findings align with earlier research (Bianco et al., 2010; Lonigan, 2007), extending evidence that structured, phonologically oriented activities can yield durable gains beyond immediate post-intervention effects.

Notably, the results also revealed significant vocabulary gains in the phonological intervention group. In the absence of a significant between-group differences, this finding should be interpreted cautiously and does not allow for firm conclusions regarding the relative contribution of phonological training to vocabulary development comparable to those observed following semantic training. Drawing on hypotheses that link vocabulary activation to morphophonological structure (Best, 2005; Dell, 1986; German, 2002; German & Newman, 2004; Levelt et al., 1999; Mengisidou et al., 2020; Roelofs, 1997; Sheng & McGregor, 2010), this intervention program explored whether strengthening phonological organization alone might be associated with vocabulary growth in Greek.

One possible explanation is that improvements in auditory discrimination and phonological encoding may have supported the consolidation of more stable phonological representations which in turn could facilitate lexical access, consistent with Bishop et al.’s (2012) assertion that accurate initial representations are essential for successful word learning. Furthermore, the highly productive morphological system of Greek—rich in derivational morphology and compounding, which allow the formation of new words through suffixation and the combination of stems or words—may have facilitated the integration of morphophonemic structure with newly acquired semantic information. Vocabulary gains in this group, however, were not sustained at the four-month follow-up, indicating that long-term retention may require additional support.

### 4.2. Semantic Intervention

The semantic intervention combined word categorization, definitions, and narrative activities to provide a comprehensive approach to semantic development. Encouragingly, in line with our expectations, children receiving semantic intervention demonstrated significant vocabulary gains, maintained three months post intervention. Emphasizing thematic (associative) and taxonomic (feature-based) categorization, along with exposure to vocabulary across multiple contexts, promoted robust learning and strengthened mental lexicon storage and retrieval. This finding aligns with previous literature linking vocabulary growth to strengthened semantic connections (Axpe et al., 2017; Ebbels et al., 2012; Hadley et al., 2019; Kelley, 2017; Wilson et al., 2015).

Despite vocabulary gains, the semantic group did not show significant improvement in phonological awareness tasks. In the absence of significant effects, these findings do not provide evidence that semantic gains alone were associated with changes in phonological awareness at this developmental stage. This contrasts with some previous findings (Beckman et al., 2007; Lonigan, 2007) suggesting that the bidirectional relationship between semantics and phonology may not hold for preschool Greek-speaking children with DLD. The limited effects observed in the present study do not appear to align with the predictions of the LRH. A possible explanation is that the young age of the study’s participants, along with their DLD status, may not have allowed sufficiently rapid vocabulary growth to exert a strong influence on phonemic awareness skills. This interpretation is supported by Verhoeven et al. (2011), who questioned this hypothesis based on evidence from illiterate adults with adequate vocabularies, arguing that semantic growth may play a facilitating role but is not sufficient to improve phonological awareness. According to their findings, vocabulary is more strongly associated with reading comprehension than with word decoding, which relies primarily on phonemic processing skills. However, the absence of evidence for lexical restructuring processes in the present study should not be construed as direct evidence of developmental constraints in young children with DLD, as this interpretation remains tentative.

### 4.3. Limitations

Despite the strengths of the present study, several limitations should be acknowledged. First, the participants were drawn from a single small city in the Greek periphery. Although specific demographic criteria were applied, the findings may not generalize to larger populations or other geographic regions of Greece. In addition, the relatively small sample size within each experimental group limits the extent to which the results can be considered representative of the broader population of preschool children. Future studies should replicate this experimental design with larger and more diverse samples.

Also, the study design did not include a second delayed assessment for the control group at four months. This was due to ethical considerations, as the control group received the second phase of the intervention after the first post-test, preventing a long-term comparison between experimental and control groups.

Finally, the interventions were conducted individually within the public-preschool settings. Intervention conditions were not always ideal, as there were instances when the school community was engaged in rehearsals, recreational activities, extended breaks, or other circumstances that could limit participants’ attention and focus. These factors likely influenced the implementation conditions of the interventions, which would have been more strictly controlled in academic or clinical settings.

### 4.4. Clinical and Research Implications

Preschool children with DLD, despite their language difficulties, can achieve meaningful improvements when provided with appropriate, targeted interventions. This study provides evidence that both phonological and semantic interventions can yield meaningful improvements, though the effects differ by domain.

The study’s findings also suggest potential complementary pathways for vocabulary development: phonological interventions enhance both phonological and vocabulary skills, but with less lasting effect on vocabulary alone, whereas semantic interventions produce more sustained vocabulary gains. The partial loss of gains in the phonological group may suggest a possible role for integrating semantic reinforcement to support long-term outcomes.

Although these findings should be interpreted cautiously given the relatively small sample size and the geographically limited participant pool, the clinical implications that may be drawn from the present results indicate that interventions combining phonological and semantic elements may be worth consideration in addressing the heterogeneous language profiles of preschool children with DLD and in supporting more favorable language outcomes. Accordingly, such combined approaches could be considered in intervention planning, with any potential benefits interpreted cautiously. Based on the structure and outcomes of the present study, a staged implementation may be useful in clinical or preschool intervention settings. An initial phase of approximately 6–8 weeks could prioritize phonological awareness activities followed by, or combined with, a semantic enrichment phase of similar duration, focusing on explicit vocabulary instruction, semantic categorization, etc., in narrative or play-based activities.

From a research perspective, these findings call for further investigation of the interaction between phonological and semantic processes in non-English languages, particularly in relation to the Lexical Restructuring Hypothesis. Future studies should explore optimal intervention intensity, duration, and balance to maximize long-term language outcomes for children with DLD.

## Figures and Tables

**Table 1:** Study timeline.

T1 Pre- Assessment	Intervention Phase 1 (16 Biweekly Sessions -8 Weeks)	Break 4 Weeks	Intervention Phase 2 (16 Biweekly Sessions -8 Weeks)	T2 Post- Assessment (5 Months Post T1)	T3 Follow-up Assessment (4 Months Post T2)
Group 1 *n* = 35	Semantic		Semantic	1. Semantic group	1. Semantic group
Group 2 *n* = 35	Phonological		Phonological	2. Phonological group	2. Phonological group
Group 3 *n* = 37	Business as usual		Business as usual	3. Control group	

**Table 2:** Distribution of participants by intervention type, sex, and age group.

Sex	Age Group (Years)	Semantic N (%)	Phonological N (%)	Control N (%)	Boys vs. Girls
Boys	4–5	10 (52.6)	9 (47.4)	8 (53.3)	χ^2^ (1) = 0.32, *p* = 0.9
	5–6	9 (47.4)	10 (52.6)	7 (46.7)	
Girls	4–5	7 (43.8)	7 (43.8)	12 (54.5)	χ^2^ (1) = 0.48, *p* = 0.7
	5–6	9 (56.3)	9 (56.3)	10 (45.5)	
Total		35	35	37	

**Table 3:** Average (SD) scores on the key cognitive and language indices of the study by intervention group at baseline (T0).

	Groups			Group Comparisons (*p* Values)		
	Semantic	Phonological	Control	Semantic vs. Phonological	Semantic vs. Control	Phonological vs. Control
Non-verbal IQ (%tile; Raven’s)	72.55 (16.90)	70.35 (17.66)	64.32 (13.03)	0.9	0.1	0.3
General Language Index	13.43 (10.93)	16.14 (12.02)	14.35 (9.72)	0.9	0.9	0.9
Phonological awareness	24.46 (20.37)	24.91 (15.64)	25.59 (22.33)	0.9	0.9	0.9
Semantic skills	19.43 (13.48)	18.83 (11.48)	18.32 (12.38)	0.9	0.9	0.9

*p* values are for *t*-tests for independent samples. Semantic vs. phonological group: |t|(68) = 0.11 to 0.99; semantic vs. control group: |t|(70) = 0.2 to 1.75; phonological vs. control group: |t|(70) = 0.15 to 1.65.

**Table 4:** Average (SD) phonological awareness scores across three time points by intervention groups.

**(A) Within-group comparisons (paired-samples *t*-tests)**							
**Group**	**T0**	**T1**	**T2**		**T0 vs. T1 (*p*)**	**T0 vs. T2 (*p*)**	**T1 vs. T2 (*p*)**
Phonological	24.91 (15.64)	44.21 (19.74)	41.60 (19.90)		<0.001	<0.001	0.5
Semantic	24.46 (20.37)	36.86 (20.85)	36.40 (19.26)		0.002	0.007	0.9
Control	25.59 (22.33)	27.45 (23.85)	-		0.50	-	-
**(B) Between-group comparisons (independent-samples *t*-tests)**							
		**T0 (*p*)**		**T1 (*p*)**		**T2 (*p*)**	
Phonological vs. Semantic		0.9		0.4		0.2	
Phonological vs. Control		0.9		0.004		-	
Semantic vs. Control		0.9		0.2		-	

Note. Dashes (“-”) indicate comparisons which were not computed due the fact that T2 data were not available for the control group.

**Table 5:** Average scores (SD) on semantic skills across three time points by intervention group.

**(A) Within-group comparisons (paired-samples *t*-tests)**							
**Group**	**T0**	**T1**	**T2**	**T0 vs. T1 (*p*)**		**T0 vs. T2 (*p*)**	**T1 vs. T2 (*p*)**
Phonological	18.83 (11.48)	33.57 (20.06)	27.38 (19.37)	<0.001		0.005	0.004
Semantic	19.43 (13.48)	34.27 (20.13)	31.39 (19.38)	<0.001		<0.001	0.3
Control	18.32 (12.38)	22.23 (15.64)	-	0.15		-	-
**(B) Between-group comparisons (independent-samples *t*-tests)**							
		**T0 (*p*)**			**T1 (*p*)**	**T2 (*p*)**	
Phonological vs. Semantic		0.9			0.9	0.4	
Phonological vs. Control		0.9			0.034	-	
Semantic vs. Control		0.9			0.022	-	

Note. Dashes (“-“) indicate comparisons which were not computed due the fact that T2 data were not available for the control group.

## Data Availability

The datasets presented in this article are not readily available due to ethical and privacy restrictions. Requests for access to anonymized data may be directed to the corresponding author and will be considered in accordance with institutional ethical guidelines.

## Supplementary Materials

The following supporting information can be downloaded at https://www.mdpi.com/article/10.3390/bs16050809/s1. Table S1: Intervention content.
