# Supplementary material for: Bridging the Gap Between Language and Literacy: Evidence from Interventions in Young Greek-Speaking Children with Developmental Language Disorder
Source: Behav Sci (Basel). 2026 May 18;16(5):809. doi: 10.3390/bs16050809 (PMC13203354; doi:10.3390/bs16050809)
Supplement: Supplementary file 1 [file behavsci-16-00809-s001.zip › behavsci-4161144-supplementary.pdf]

Table S1. Intervention content

**Semantic intervention**	**Phonological intervention**
Word meanings - comprehension<br>*The child identifies an object named (e.g., "Put a bandage on the baby's knee.").*	Syllable blending and segmentation<br>*The child blends syllables to form a word or segments a word into syllables (e.g., /ba/ – /na/ – /na/ → banana; or banana → /ba/ – /na/ – /na/).*
Word categorization based on thematic relations<br>*The child associates two objects that are thematically related (e.g., dress – skirt).*	Identification of initial syllables in words<br>*The child identifies the first syllable of a word (e.g., table → /ta/)*<br>Discrimination of initial syllables across words<br>*The child determines whether two words begin with the same syllable (e.g., pencil – penguin vs pencil – tiger).*
Word categorization based on categorical relations – perceptual/visual features<br>*The child sorts objects according to their perceptual or visual characteristics (e.g., toys with wheels vs without wheels).*	Identification of medial syllables in words<br>*The child identifies the middle syllable in a multisyllabic word (e.g., tomato → /ma/)*<br>Identification of final syllables in words<br>*The child identifies the last syllable of a word (e.g., paper → /per/).*<br>Discrimination of final syllables across words<br>*The child determines whether two words end with the same syllable (e.g., window – pillow vs window – table).*
Word categorization based on categorical relations – defining taxonomic features<br>*The child categorizes items according to taxonomic characteristics (e.g., classifying fruits and vegetables based on features such as peel/skin and seeds).*	Word generation based on initial syllables<br>*The child produces words that begin with a given syllable (e.g., /pa/ → panda, paper, pasta).*
Semantic associations – metacognitive reflection<br>*The child is asked to consider similarities and differences between items (e.g., wolf – fox).*	Rhyme detection / identification of rhyming words<br>*The child identifies which words rhyme (e.g., cat – hat vs cat – cup).*
Semantic analogies<br>*The child matches related pairs (e.g., head – hat, eyes – glasses).*	Identification of initial phonemes in words<br>*The child identifies the first sound in a word (e.g., sun → /s/).*<br>Discrimination of initial phonemes across words<br>*The child determines whether two words begin with the same sound (e.g., ball – book vs ball – dog).*
Sentence completion<br>*The child listens to a sentence and completes it with the target word (e.g., "I hang my bag on my … (shoulders).")*	Identification of medial phonemes in words<br>*The child identifies the middle sound in a simple word (e.g., cat → /a/).*<br>Identification of final phonemes in words<br>*The child identifies the last sound in a word (e.g., dog → /g/).*<br>Discrimination of final syllables across words<br>*The child decides which words share the same final syllable (e.g., basket – rocket vs basket – candle).*
Word descriptions – definitions<br>*The child listens to a short description and identifies the target word (e.g., "We wear them on our legs in winter, and they are long.").*	Word generation based on initial phonemes<br>*The child produces words that begin with a given phoneme (e.g., /m/ → moon, milk, mouse).*
Narrative production<br>*The child is asked to create a story using the words introduced in each subsection.*	Manipulation of word parts (addition–deletion of syllables and phonemes) *The child adds or deletes syllables or phonemes to form new words (e.g., say "play" without /p/ → lay; add /s/ to mile → smile; remove /ba/ from banana → nana).*
